# Thawing Frozen Robust Multi-array Analysis (fRMA)

**DOI:** 10.1186/1471-2105-12-369

**Published:** 2011-09-16

**Authors:** Matthew N McCall, Rafael A Irizarry

**Affiliations:** 1 Department of Biostatistics and Computational Biology, University of Rochester Medical Center, Rochester, NY, USA; 2 Department of Biostatistics, Johns Hopkins University, 615 North Wolfe Street, Baltimore, MD, USA

## Abstract

**Background:**

A novel method of microarray preprocessing - Frozen Robust Multi-array Analysis (fRMA) - has recently been developed. This algorithm allows the user to preprocess arrays individually while retaining the advantages of multi-array preprocessing methods. The *frozen *parameter estimates required by this algorithm are generated using a large database of publicly available arrays. Curation of such a database and creation of the frozen parameter estimates is time-consuming; therefore, fRMA has only been implemented on the most widely used Affymetrix platforms.

**Results:**

We present an R package, frmaTools, that allows the user to quickly create his or her own frozen parameter vectors. We describe how this package fits into a preprocessing workflow and explore the size of the training dataset needed to generate reliable frozen parameter estimates. This is followed by a discussion of specific situations in which one might wish to create one's own fRMA implementation. For a few specific scenarios, we demonstrate that fRMA performs well even when a large database of arrays in unavailable.

**Conclusions:**

By allowing the user to easily create his or her own fRMA implementation, the frmaTools package greatly increases the applicability of the fRMA algorithm. The frmaTools package is freely available as part of the Bioconductor project.

## 1 Background

In microarray data analysis, the process of converting probe-level flourescent intensities from a scanner to gene-level expression estimates is commonly referred to as *preprocessing*. The vast majority of preprocessing algorithms require multiple arrays to be analyzed simultaneously, and in general such *multi-array *preprocessing algorithms outperform single-array algorithms [1]. Therefore, it is not surprising that four of the most widely used preprocessing algorithms - RMA [2], gcRMA [3], MBEI [4], and PLIER [5] - are multi-array.

However, multi-array preprocessing algorithms restrict scientific inquiry because it is necessary to analyze all arrays simultaneously. Because data preprocessed separately cannot be combined without introducing artifacts [6-10], the total number of arrays one can compare is limited by computer memory, restricting large meta-analyses; furthermore, datasets that grow incrementally need to be preprocessed each time an array is added. Lastly, for microarrays to aid in clinical diagnosis and treatment, one needs to obtain information based on a single sample hybridized to a single microarray.

Recent work by McCall et al. (2010) provided a method of single-array preprocessing, Frozen Robust Multiarray Analysis (fRMA), that retains the advantages of multi-array preprocessing. The fundamental idea behind fRMA is a simple but powerful one - parameter estimates are precomputed based on a large biologically diverse database of microarrays and then *frozen*. These frozen parameter estimates can then be used to preprocess new array(s). In addition to allowing arrays to be preprocessed individually or in small batches and combined for subsequent analysis, fRMA resulted in improved performance when analyzing microarray data in the typical multi-array setting, especially when the new data consisted of arrays from different batches [10].

It should be noted that fRMA allows the user to easily combine Affymetrix microarrays from different batches; however, these arrays must all be from the same platform. For example, fRMA cannot be used to preprocess Affymetrix Human Genome U133A and U133 Plus 2.0 arrays together. To combine different platforms, one would need to first preprocess the data from each platform separately using fRMA and then combine the data using an additional batch correction algorithm such as ComBat [11] or Surrogate Variable Analysis (SVA) [12].

The primary limitation of fRMA is that it requires a curated database of microarrays to generate the frozen parameter vectors. As such, fRMA has currently been implemented for only the three most widely used Affymetrix microarray platforms - Human Genome U133 Plus 2.0, Human Genome U133A, and Mouse Genome 430 2.0. Efforts are underway to implement fRMA on two more platforms - Arabidopsis ATH1 Genome and Rat Genome 230 2.0.

To address this limitation, we have created an R package, frmaTools, designed to allow the user to easily create the frozen parameter vectors necessary to run fRMA. By decentralizing fRMA implementation, each group can contribute a database and parameter estimates specific to their area of research. We begin by describing the frmaTools package itself and how it interfaces with the standard frma package. Next we examine the size and scope of data necessary for a platform-wide implementation of fRMA. We then turn our attention to situations in which one might want to generate custom parameter vectors for a specific experiment.

## 2 Implementation

The computational tools described here are written in the open-source statistical language R and are available as part of the Bioconductor project [13], a collaborative effort to produce computational tools for biological data. As previously described in [10], we have implemented the primary tools necessary to preprocess and analyzed data from a single microarray hybridization in the R package, frma. This package provides the fundamental framework for single array preprocessing and analysis; however, it requires precomputed frozen parameter vectors. For platforms on which fRMA has been already implemented these frozen parameter vectors are contained in Bioconductor data packages of the form *<*platform*>*frmavecs and are automatically used by frma. To extend the fRMA algorithm to a new platform, one need only create a data package or supply ones own frozen parameter vectors directly to frma.

To preprocess single arrays using fRMA, one needs 5 frozen parameter vectors: (1) the reference normalization distribution, (2) the probe effect estimates, (3) the within batch residual variance, (4) the between batch residual variance, and (5) the within probeset average standard deviation. Optionally, a sixth vector of median standard errors is necessary to compute a measure of array quality. By default, the frma function attempts to load the appropriate data package for the AffyBatch object given as input. However, it is possible for the user to supply some or all of the frozen parameter vectors allowing the user to preprocess data using their own fRMA implementation.

**Figure 1:**
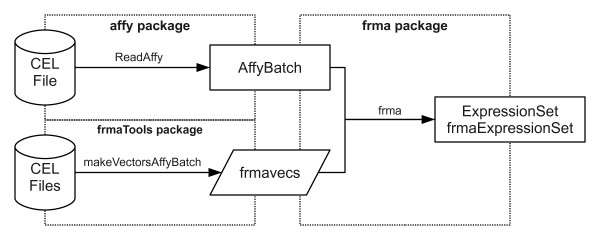
**Diagram of a typical workflow**. A typical workflow using the affy, frma, and frmaTools packages to obtain fRMA gene expression estimates in the form of an ExpressionSet or frmaExpressionSet object. Above each arrow is the function used to transform one object into the next. The dashed lines divide the figure by the package used.

The frmaTools package was developed to allow users to easily create their own frozen parameter vectors for use with frma. It contains two primary functions - makeVectorsAffyBatch and makeVectorPackage. The former is used to create the frozen parameter vectors used by frma; the latter both creates the vectors and builds a R data package for them. By creating a data package, one can share frozen parameter vectors with other researchers and track changes over time. The interface between the affy, frma, and frmaTools packages can best be understood by examining a typical workflow (Figure 1).

## 3 Results and Discussion

### 3.1 Training Data

The frozen parameter vectors used in the fRMA algorithm are generated using a training dataset comprised of a balanced sample from multiple batches. For example, in the three current implementations of fRMA, 5 arrays from each of 200 different batches are used. In this case, batch is defined as a unique combination of tissue type and experiment id (e.g. normal liver from GSE2004). Here the between batch variability in probe effects captures all the potential differences between experiments - different labs, different technicians, different environmental conditions, etc. - as well as differences due to the hybridized tissue type.

One of the fundamental assumptions underlying fRMA is that the training data is both large enough and diverse enough that the vast majority of probes that differ in performance across batches can be detected as such in the training data. Some probes may only perform poorly in a small handful of tissues or laboratory conditions, so it is advantageous to include as many different tissues and experiments as possible to maximize our chance of detecting unreliable probes. When implementing fRMA on widely used platforms for which there is a plethora of publicly available data, it is advisable to use as many arrays as possible. This allows one to obtain the most information about the behavoir of probes across a wide variety of tissues and experiments. In these situations, the size of the training dataset should only be constrained by computer memory.

For a fixed amount of memory corresponding to a fixed number of arrays in the training dataset, the primary question is whether it is better to include a greater number of batches (and thereby fewer arrays per batch) or a greater number of arrays per batch (and thereby fewer batches). To address this question, we examined a variety of training datasets of varying number of batches and batch size. For each combination we repeated the random sampling used to select the training arrays 100 times. For each set of parameter vectors, we preprocessed a single average-quality array (GSM282373) and computed the median absolute deviation (MAD) across the 100 replicates for each probeset. To assess the consistency of fRMA for a variety of training datasets, we report the median and inter-quartile range (IQR) of the within-gene MADs (Table 1).

**Table 1:** Training data size affects fRMA reproducibility

		Batch Size				
		**3**	**5**	**10**	**15**	**20**

**Number of Batches**	5	0.7585 (0.1574)	0.7573 (0.1437)	0.8515 (0.1435)	0.5812 (0.1231)	0.4439 (0.0916)
	10	0.6795 (0.0799)	0.7173 (0.0858)	0.6563 (0.0641)	0.4506 (0.1073)	0.3878 (0.0901)
	20	0.5696 (0.0654)	0.4691 (0.0523)	0.5180 (0.0506)	0.4551 (0.0561)	0.3299 (0.0629)
	30	0.4429 (0.0491)	0.3884 (0.0482)	0.3387 (0.0380)	0.3697 (0.0537)	0.3036 (0.0440)
	40	0.3290 (0.0450)	0.3700 (0.0488)	0.2598 (0.0368)	0.2642 (0.0303)	
	50	0.3093 (0.0424)	0.3107 (0.0339)	0.2307 (0.0291)		
	60	0.2661 (0.0374)	0.2454 (0.0322)	0.1955 (0.0261)		
	70	0.2529 (0.0322)	0.2286 (0.0295)	0.2089 (0.0261)		
	80	0.2256 (0.0281)	0.2098 (0.0285)	0.1616 (0.0259)		
	90	0.1922 (0.0274)	0.2058 (0.0248)	0.1566 (0.0163)		
	100	0.1891 (0.0277)	0.1976 (0.0261)	0.1128 (0.0167)		

Median and IQR of the across-replicate median absolute deviations (MAD) for different batch sizes (columns) and number of batches (rows) used to train the fRMA algorithm. The median provides an estimate of the typical MAD; the IQR provides an estimate of the variability seen in MADs across replicates.

As one would expect, increasing the number of batches while holding batch size constent, typically improves the consistency of the results. However, increasing batch size while holding the number of batches constant appears to result in increased consistency in general, but this increase is far less reliable than increasing the number of batches. Specifically, increasing the batch size from 3 to 5 is just as likely to decrease consistency as increase it. However, increasing batch size from 5 to 10 or 10 to 15 yields increased consistency more often than not, and increasing batch size from 15 to 20, results in increased consistency for all batch sizes examined. This suggests that consistency is driven primarily by the ability to detect probes whose behavior differs between batches rather than detection of probes that are highly variable within batches and that detection of highly variable probes within batches requires a sizable investment in terms of the number of arrays per batch. Finally, the improvements in consistency due to adding additional batches, seem to diminish as the number of batches increases. Taken together these findings suggest that, one should first seek to increase the number of batches and then the number of samples per batch.

However, for future fRMA implementations, computer memory may not be the limiting factor. For newer microarray platforms, it is often the case that a relatively small number of labs have conducted one or more large experiments possibly involving a wide variety of tissues. As Table 1 suggests, this is not the ideal situation and training fRMA on such data may not capture the behavior of a number of probes. In such a situation, it may be more appropriate to consider a custom fRMA implementation for either a single large dataset or a single lab. Such an implementation will be described in Section 3.4.

### 3.2 Alternative CDF

Affymetrix microarray platforms by default use probe target definitions based on the knowledge available when they were created. Since then, our understanding of the human and mouse transcriptomes has greatly improved. Multiple studies have demonstrated that using updated probe annotations can significantly improve gene expression estimates and detection of differential expression [14-16].

While the original implementation of fRMA was based on the default Affymetrix probe annotation, the latest version of the frma package gives one the option to use the version 13 Entrez Gene probe annotation [14]. However, there are numerous other probe annotations that might be of interest (e.g. RefSeq, Ensembl, etc.). The frmaTools package allows one to choose any of the alternative annotations to create one's own fRMA implementation.

### 3.3 New Platform

Although we strive to implement fRMA on all Affymetrix microarray platforms for which there is a sufficient amount of publicly available data, fRMA may not currently be implemented on a platform of interest. If there is enough data publicly available, one has the option of creating one's own implementation by following the recommendations outlined in Section 3.1 and using the frmaTools package described in Section 2. However, for a relatively new microarray platform, there may not be enough publicly available data. In this case, one might consider creating a custom fRMA implementation. We describe this option in the following section.

### 3.4 Custom implementations of fRMA

The fRMA preprocessing algorithm typically requires a large diverse dataset to estimate the frozen parameters. The dataset is required to span a large number of batches in order to capture the variability in probe behavior, so that when preprocessing a new array, one can appropriately down-weight probes known to have either large between- or within-batch variance. While it is always possible that a probe that appears reliable in the training data performs poorly on a new array, the chance of this occurring can be minimized by training on a large diverse dataset.

However, a large diverse dataset is not always available either because the microarray platform is relatively new or because the data being analyzed differs from the publicly available data in some fundamental manner (e.g. a non-standard lab protocol or non-human RNA hybridized to a human array). In these cases, one might consider creating one's own custom fRMA implementation that is specific to a certain dataset, lab, or experiment.

#### 3.4.1 RMA-like implementation

If it is infeasible (due to lack of data) or undesireable (due to properties of the data) to use a standard fRMA implementation to analyze one's data, it could be beneficial to implement fRMA in a manner that mimics RMA. While the primary advantage of fRMA over RMA is the ability to preprocess a single microarray, the fRMA algorithm also improved the RMA model by recognizing that some probes within a probe set were more variable than others and down-weighting such probes [10]. For this reason, one might be interested in applying fRMA within a large dataset in the same manner one would apply RMA.

Such an implementation can easily be accomplished using the frmaTools package. To illustrate the ability of fRMA to mimic RMA when analyzing a single dataset, we compared the expression values generated by RMA to those generated by 3 different fRMA implementations:

1. the default fRMA implementation trained on a large diverse sample of arrays,

2. fRMA trained on a balanced random sample from the experiment being analyzed,

3. same as (2) but using all the arrays to form the reference normalization distribution.

**Figure 2:**
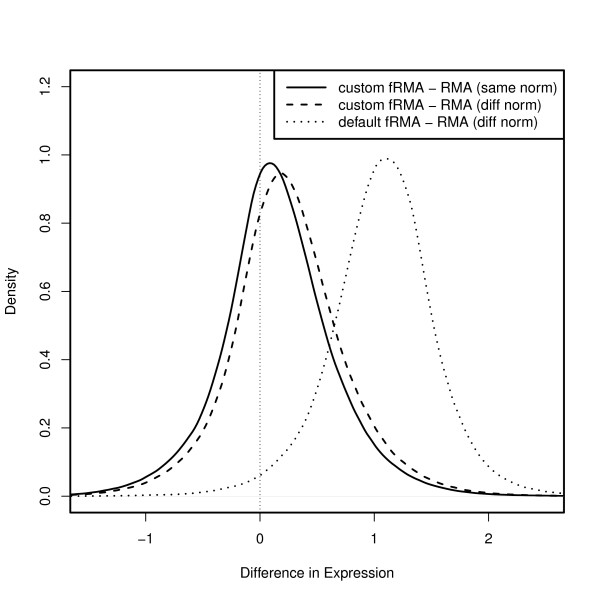
**fRMA can mimic RMA**. Distribution of difference in expression estimates for 22283 probesets across 200 breast tumor arrays (GSE11121) between 3 different fRMA implementations and RMA. The 3 fRMA implementations are as follows: (1) the default fRMA implementation (dotted line), (2) fRMA trained on a balanced random sample from the arrays being analyzed (dashed line), and (3) fRMA trained on a balanced random sample from the arrays being analyzed and using the same reference distribution for quantile normalization as RMA.

As one would expect, the differences in expression values are much greater between the default fRMA implementation and RMA than between the two custom fRMA implementations and RMA (Figure 2). It is important to assess whether the observed differences between the custom fRMA implementation and RMA are due differences in performance. To assess this, we used a large pseudo-simulated spike-in dataset generated by extending the Affymetrix Human Genome U133A spike-in dataset. Specifically, we replicated each of the original arrays 3 times to create 9 batches of 14 arrays, each with a latin square spike-in design. We then added both random noise and random batch effects to each probe based on that probe's observed behavior in a large biologically diverse dataset. This resulted in a dataset with probe-specific noise and batch effects comparable to a typical large dataset.

We used this extended spike-in dataset to assess the performance of RMA and fRMA trained on only these data. Specifically, we assessed the accuracy, precision, and overall performance of both methods in three expression strata using the methodology proposed in [17]. We assessed accuracy by computing the *signal detection slope*, the slope from regressing observed expression on nominal concentration in the *log*_2 _scale. The signal detect slope is the expected difference in observed expression when the true difference is a fold change of 2, with the optimal result being a slope of one. We assessed precision by computing the standard deviation and 99.5th percentile of the *null log-ratios*, log-ratios from transcripts which were not spiked in and therefore should not be differentially expressed. The standard deviation is an estimate of the variability in log-ratios for non-differentially expressed genes; the 99.5th percentile assesses outliers - 0.5% of non-differentially expressed genes are expected to exceed this value. Finally, we report the signal-to-noise ratio (SNR) and the probability of a gene with a true *log*_2 _fold change of 2 being in a list of the 100 genes with the highest fold change (POT).

In all three strata, fRMA outperformed RMA with regard to precision and overall performance (Table 2). In the low and medium strata, fRMA also had better accuracy than RMA; however, in the high strata, RMA and fRMA had comparable accuracy with RMA performing slightly better. The primary difference between the two preprocessing methods is that fRMA does not treat all probes as equally reliable - it down-weights probes that have high within- or between-batch variance. These results suggest that when preprocessing a fairly large dataset, it is potentially beneficial to use a custom fRMA implementation if a standard fRMA implementation is not available.

**Table 2:** Comparison of RMA and fRMA based on bias, precision, and overall performance

	Preprocessing	Slope (SD)	Null SD	Null 99.5%	SNR	POT
**Low**	RMA	0.14 (0.54)	0.47	1.46	0.30	0.01
	fRMA	0.20 (0.48)	0.40	1.24	0.50	0.02

**Medium**	RMA	0.69 (0.55)	0.39	1.26	1.77	0.15
	fRMA	0.75 (0.49)	0.34	1.11	2.21	0.23

**High**	RMA	0.61 (0.42)	0.33	0.99	1.85	0.18
	fRMA	0.60 (0.36)	0.28	0.86	2.14	0.23

Comparison of RMA and fRMA trained on the modified spike-in data. For three intensity strata, we report assessments of accuracy (column 1), precision (columns 2 & 3), and overall performance (columns 4 & 5).

#### 3.4.2 Incrementally growing, large dataset

Another reason one might wish to create a custom fRMA implementation is to analyze a dataset in which samples are added in small batches over a period of time and intermediate analysis would be beneficial. For example, suppose researchers are interested in investigating a gene signature for breast cancer prognosis when treated with a novel chemotheraputic drug. At the start of treatment, a biopsy of each patient's breast tumor is hybridized to a single microarray. As the results begin to trickle in, the researchers are able to train the fRMA model based on the currently available data and use that model to predict the response of patients currently undergoing treatment.

In contrast, most multi-array preprocessing methods would require the entire dataset to be preprocessed each time a new array was added. This poses several potential problems. First, although preprocessing hundreds of arrays with RMA requires relatively little computational time, other multi-array preprocessing methods take significantly longer. Second, while RMA requires little computational time, it does require large amounts of memory - preprocessing 200 arrays requires over 11GB of RAM; for 500 arrays, this increases to over 30GB. Third, once an array has been preprocessed with fRMA, it's gene expression estimates will remain the same regardless of any additional arrays that are added to the data. The same cannot be said of multi-array preprocessing methods, which would provide different expression estimates for a given array each time an additional array is added and the data are preprocessed again.

To assess the applicability of the fRMA algorithm in such a situation, we consider a dataset of 200 breast tumor samples (GSE11121) collected from patients over 477 days from Jan 29th, 2004 to March 20th, 2005. The arrays were grouped in 13 batches by the date on which the array was scanned. We defined a batch as consecutive dates over which at least 5 arrays were hybridized. Only 16 out of the 200 arrays did not fall into a batch.

**Figure 3:**
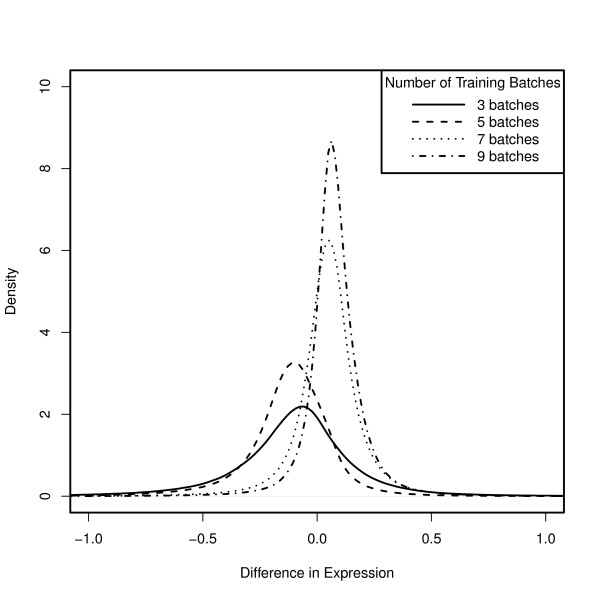
**fRMA can be used with incrementally growing datasets**. Distribution of difference in expression estimates for 22283 probesets across 200 breast tumor arrays (GSE11121) when preprocessing with fRMA trained using the first N batches and fRMA trained using all batches.

**Figure 4:**
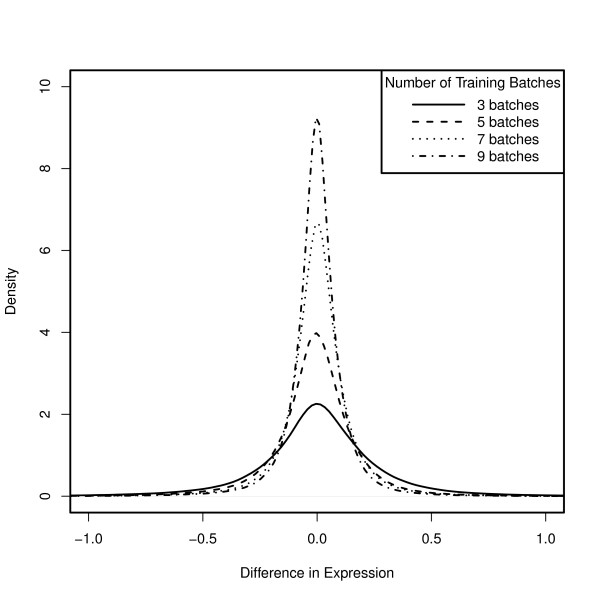
**Using the same normalization vector reduces bias**. Same as Figure 3 except that the same normalization vector is used for all of the implementations. This suggests that differences in the center of the distributions are primarily due to differences in the reference distribution used in quantile normalization and that differences in the spread of the distributions are primarily due to estimation of the probe effects.

First, we trained the fRMA algorithm using 5 arrays randomly selected from each of the 13 batches and stored the resulting parameter vectors. We then trained the fRMA algorithm using only the first N batches where we let N vary from 3 to 9. We then used each of these sets of parameter vectors to preprocess all 200 arrays. Finally, we subtracted the expression estimates based on all 13 batches from the expression estimates based on each of the other subsets. As one would expect, the distributions of these differences are centered close to zero and their spread decreases as the number of training batches increases (Figure 3). The bias, deviation of the center of the distribution from zero, can be explained primarily by the quantile normalization reference distribution. In fact, if we quantile normalize to a fixed reference distribution, all of the distributions are centered roughly at zero (Figure 4). On the other hand, the spread of the distribution is primarily due to the summarization step, in particular, the probe effect estimation. From this example, we can also gain insight into the number of batches one should include in the training data for a custom fRMA implementation. While training on the first 3 batches produced expression estimates that differed the most from training on the full dataset, the differences were actually remarkably small - 1.6% of the absolute differences exceeded 1, and 9.4% exceeded 0.5, with the median absolute difference equal to 0.15. If instead one were to train on roughly the first half of the data (the first 7 batches), only 0.2% of the absolute differences exceeded 1, and 2% exceeded 0.5, with the median absolute difference equal to 0.08. This suggests that training on just the first 3 batches would allow one to detect moderate to large changes in gene expression and that training on the first half of the data, perhaps from an initial pilot study, would be enough to detect relatively small changes in gene expression.

#### 3.4.3 A different definition of batch

In the current implementations of fRMA, batch is defined as the combination of tissue and experiment. However, there are certainly batch effects present within many experiments [18]; therefore, it could be advantageous to define batch in a different way. For example, in Section 3.4.2 we defined batch based on the date on which an array was scanned. There are many other potential sources of variation in an experiment any of which could be used to investigate batch effects. The frmaTools package allows the user the flexibility to define batch in any way he or she desires.

In fact, even if the user eventually plans to use a preprocessing method other than fRMA, examining the frozen parameter vectors generated by frmaTools may provide insights in to batch effects present in the data that need to be addressed in some manner. Specifically, we can compute an F-statistic for each probe, defined as the ratio of between-batch and within-batch variance, to assess whether that probe displays a batch effect in a given data set. To illustrate this, we computed these ratios for the data presented in Section 3.4.1 and the data presented in Section 3.4.2. In the former case, 20.3% of probes show a statistically significant batch effect, while in the latter case, only 1.8% of probes show a batch effect. Thus by simply comparing the within- and between-batch probe variances produced by frmaTools, we can assess the extent of batch effects within a given dataset.

## 4 Conclusions

We have described a novel R package, frmaTools, that allows the user to easily create his or her own fRMA implementation. We have described how this new package fits into a standard analysis workflow and the size and scope of data necessary to make use of it. This package will allow researchers to use fRMA on a much wider range of datasets and annotations. Furthermore, we have described several situations in which a dataset-specific custom fRMA implementation might be advantageous. The frmaTools package makes such custom implemenations straightforward. The frmaTools package is freely available as part of the Bioconductor project and as Additional File 1.

## Competing interests

The authors declare that they have no competing interests.

## Availability and requirements

• **Project name: **frmaTools

• **Project home page: **http://www.bioconductor.org/packages/release/bioc/html/frmaTools.html

• **Operating system(s): **Platform independent

• **Programming language: **R

• **Other requirements: **R *>*= 2.10

• **License: **GNU GPL

• **Any restrictions to use by non-academics: **None

## Authors' contributions

MNM conceived the study, created the R package, and wrote the manuscript. RAI conceived the study and edited the manuscript. All authors read and approved the final manuscript.

## Supplementary Material

Additional file 1 **frmaTools package**. The R/Bioconductor package frmaTools (version 1.4.0), providing tools for advanced use of the frma package. Click here for file
