# Supplementary material for: Thawing Frozen Robust Multi-array Analysis (fRMA)
Source: BMC Bioinformatics. 2011 Sep 16;12:369. doi: 10.1186/1471-2105-12-369 (PMC3180392; doi:10.1186/1471-2105-12-369)
Supplement: Additional file 1 — frmaTools package. The R/Bioconductor package frmaTools (version 1.4.0), providing tools for advanced use of the frma package. [file 1471-2105-12-369-S1.GZ › frmaTools/inst/doc/frmaTools.pdf]

Tools for Advanced Use of the frma Package (frmaTools)

Matthew N. McCall

April 14, 2011

Contents

1 Introduction **1**

2 Creation of Vectors **2**

3 Platform Conversion **3**

1 Introduction

Frozen RMA (fRMA) is a microarray preprocessing algorithm that allows one to analyze microarrays individually or in small batches and then combine the data for analysis. This is accomplished by utilizing information from the large publicly available microarray databases. In particular, estimates of probe-specific effects and variances are precomputed and frozen. Then, with new data sets, these are used in concert with information from the new array(s) to normalize and summarize the data.

This document describes frmaTools, which provides a few advanced usage options above and beyond the basic frma functionality. Details of the ideas implemented in the frma and frmaTools packages can be found in: *McCall, MN, Bolstad, BM, and Irizarry, RA (2010). Frozen robust multiarray analysis (fRMA), Biostatistics, doi: 10.1093/biostatistics/kxp059.* Ideally someone interested in using this package would first read that paper and then proceed to the sections below.

2 Creation of Vectors

To create the vectors required by the package frma, one needs a large database of biologically diverse samples from a variety of batches. For this reason, we have attempted to create and supply a number of these collections of vectors through packages available on the Bioconductor website.

To create custom vectors of one's own, follow these steps:

1. Download and install frmaTools
2. Create a directory and move all the relevant *CEL* files to that directory.
3. If using linux/unix, start R in that directory.
4. If using the Rgui for Microsoft Windows make sure your working directory contains the *CEL* files (use "File -> Change Dir" menu item).
5. Load the library.

   ```
   > library(frmaTools)
   ```

6. Create a vector of CEL file names. If your directory contains only CEL files, do:

   ```
   > files <- dir()
   ```

7. Create batch ids based on your arrays. There needs to be an equal number of arrays in each batch.
8. Run makeVectorsAffyBatch

   ```
   > vectors <- makeVectorsAffyBatch(files, batch.id)
   ```

This will create a list with the required vectors:

normVec normalization reference vector

probeVec probe effect vector

probeVarWithin within batch probe variance

probeVarBetween between batch probe variance

probesetSD within probeset standard deviation

medianSE median standard error of gene expression estimates

3 Platform Conversion

Occasionally it will be beneficial to convert microarray data from one platform to another. This is possible in cases where the probes on one platform are a subset of the probes on another platform. For example, hgu133a is a subset of hgu133atag; therefore, it is possible convert hgu133atag data to hgu133a data by discarding those probes that are present on hgu133atag but not hgu133a.

Steps to convert an AffyBatch object:

1. Download and install frmaTools

2. Load the library.

   ```
   > library(frmaTools)
   ```

3. Load the AffyBatch object to convert (for example hgu133atag data)

   ```
   > library(frmaExampleData)
   > data(list = "AffyBatch133atag")
   ```

4. Run convertPlatform (for example convert to hgu133a)

   ```
   > object <- convertPlatform(AffyBatch133atag, "hgu133a")
   ```
